# Fabrication, Characterization and Biomedical Evaluation of a Statistically Optimized Gelatin Scaffold Enriched with Co-Drugs Loaded into Controlled-Release Silica Nanoparticles

**DOI:** 10.3390/molecules28135233

**Published:** 2023-07-05

**Authors:** Hina Younis, Hafeez Ullah Khan, Safirah Maheen, Malik Saadullah, Shahid Shah, Nabeel Ahmad, Sameer Alshehri, Mohammed Ali A. Majrashi, Abdullah Alsalhi, Rida Siddique, Mehwish Andleeb, Saleha Shabbir, Ghulam Abbas

**Affiliations:** 1 Department of Pharmaceutics, College of Pharmacy, University of Sargodha, Sargodha 40100, Pakistan; hinayounis219@gmail.com (H.Y.); qarani_pharmacist@yahoo.com (H.U.K.); drmehwishandleeb@ymail.com (M.A.); salehashabbir909@gmail.com (S.S.); 2 Department of Pharmaceutical Chemistry, Faculty of Pharmaceutical Sciences, Government College University Faisalabad, Faisalabad 38000, Pakistan; maliksaadullah@gcuf.edu.pk; 3 Department of Pharmacy Practice, Faculty of Pharmaceutical Sciences, Government College University Faisalabad, Faisalabad 38000, Pakistan; shahid.waris555@gmail.com; 4 School of Chemical and Materials Engineering, National University of Science and Technology, Islamabad 44000, Pakistan; nabeel.ahmed@scme.nust.edu.pk; 5 Department of Pharmaceutics and Industrial Pharmacy, College of Pharmacy, Taif University, Taif 21944, Saudi Arabia; s.alshehri@tu.edu.sa; 6 Department of Pharmacology, College of Medicine, University of Jeddah, Jeddah 23890, Saudi Arabia; mamajrashi@uj.edu.sa; 7 Department of Pharmaceutics, College of Pharmacy, Jazan University, Jazan 45142, Saudi Arabia; aalsalhi@jazanu.edu.sa; 8 Department of Pharmacology, Faculty of Pharmaceutical Sciences, Government College University Faisalabad, Faisalabad 38000, Pakistan; ridasiddique@rocketmail.com; 9 Department of Pharmaceutics, Faculty of Pharmaceutical Sciences, Government College University Faisalabad, Faisalabad 38000, Pakistan

**Keywords:** nanoparticles, scaffolds, triamcinolone acetonide, econazole nitrate, antifungal activity

## Abstract

The current study focused on the fabrication of a well-designed, biocompatible, physically stable, non-irritating and highly porous gelatin scaffold loaded with controlled-release triamcinolone acetonide (TA) and econazole nitrate (EN) co-loaded into mesoporous silica nanoparticles (EN-TA-loaded MSNs) to provide a better long-lasting antifungal therapeutic effect with minimal unfavorable effects. Optimization of the MSNs-loaded scaffold was performed using central composite rotatable design (CCRD), where the effect of gelatin concentration (X1), plasticizer (X2) and freezing time (X3) on the entrapment of EN (Y1) and TA (Y2) and on the release of EN (Y3) and TA (Y4) from the scaffold were studied. The significant compatibility of all formulation ingredients with both drugs was established from XRD, DSC and FT-IR spectra analyses while SEM and zeta studies represented a very precise unvarying distribution of the loaded MSNs in the porous structure of the scaffold. The stability of the optimized scaffold was confirmed from zeta potential analysis (−16.20 mV), and it exhibited higher entrapment efficiency (94%) and the slower (34%) release of both drugs. During in vitro and in vivo antifungal studies against *Candida albicans*, the MSNs-loaded scaffold was comparatively superior in the eradication of fungal infections as a greater zone of inhibition was observed for the optimized scaffold (16.91 mm) as compared to the pure drugs suspension (14.10 mm). Similarly, the MSNs-loaded scaffold showed a decreased cytotoxicity because the cell survival rate in the scaffold presence was 89% while the cell survival rate was 85% in the case of the pure drugs, and the MSNs-loaded scaffold did not indicate any grade of erythema on the skin in comparison to the pure medicinal agents. Conclusively, the scaffold-loaded nanoparticles containing the combined therapy appear to possess a strong prospective for enhancing patients’ adherence and therapy tolerance by yielding improved synergistic antifungal efficacy at a low dose with abridged toxicity and augmented wound-healing impact.

## 1. Introduction

The skin, being the largest tissue, covers and protects the body from the adverse effects of external stimuli such as temperature and micro-organisms [1]. Infections caused by pathogenic fungi are usually superficial and are limited to nails, hairs, mucous membranes and epidermis. However, these fungal infections may be dangerous or life-threatening if a widespread, person-to-person transmission and a high incidence rate of infection persist. Some species of even nonpathogenic fungi can cause severe complications in an immune-compromised host [2]. The antifungal drugs ketoconazole, econazole, itraconazole, voriconazole, miconazole and fluconazole are the most frequently used. These antifungal drugs in the form of creams are currently available and applied for several mycotic and dermatological infections, but numerous fungal infections are persistent, highly resistant and reverse any efforts to cure or control them. Patients who receive topical treatment may experience local side effects such as stinging, rashes, burning, erythema, soreness and skin irritation. Patients also find topical ointments less tolerable because of their short duration of action and excessive viscosity, which makes them difficult to apply to dermatitis and other skin conditions [3,4]. Further complications linked with the topically applied creams include the instability of the creams, such as the separation of phases of emulsion triggered by the salting-out phenomenon [5]. Patients with persistent or severe skin infections may need a prolonged therapy because these standard drug-delivery systems are inefficient and need substantial concentrations of active medicine to be included for effective therapy. Despite early diagnosis and appropriate antifungal treatment, clinical outcomes remained disappointing and required combination therapy.

Numerous approaches have been used to resolve the discrepancies associated with the traditional treatment modes of dermal infections; these approaches include solid lipid nanoparticles [6,7], silica nanoparticles [8,9], nano-sponges [10,11] and scaffolds [12,13]. Sustained and targeted drug delivery, being localized delivery, combines the advantage of the requirement of a lower concentration of the drugs along with a lesser effect on healthy tissues in comparison to systemic delivery. Today, scaffolds loaded with drugs are gaining much attention [14]. However, these fabricated scaffolds are intended to have the ability to release a medicinal agent in a controlled manner. Yet the direct incorporation of these medicinal agents into the scaffold could cause a burst release of drugs and the exposure of these agents to unfavorable conditions. Utilizing microcarriers as a vehicle for drug delivery ensures the protection of the drugs from the environment and grants a controlled-release property to the scaffold along with structural support [15].

Today, silica-based mesoporous nanoparticles are under the spotlight because of their established advantages in drug delivery [16]. These nanoparticles are considered as novel and promising drug-delivery systems because of their distinguishing mesoporous structure with higher chemical stability, biocompatibility and suitable surface functionalization which guarantee not only the target delivery but also the controlled release of various drug molecules [17].

A well-designed, biodegradable and biocompatible highly porous gelatin scaffold loaded with a controlled drug-delivery system such as mesoporous silica nanoparticles (MSNs) can overcome the problems of conventional drug therapy such as repetitive use, high concentration of drugs and poor drug penetration, while also providing a better therapeutic efficacy of the drugs with minimal burst or unfavorable effects. Moreover, as the gelatin scaffold comes in contact with the wound exudates, these are proved to be highly absorptive in nature and to promote healing. The gelatin scaffolds do not cause irritation to the skin, and provide a moist environment and hemostatic effects. Having low adherence, they are easy to remove from the skin when discontinuation of treatment is required.

Co-administration therapy of corticosteroids (triamcinolone acetonide) and azoles (econazole nitrate) leads to the beneficial co-delivery effects of therapy to eradicate resistant skin diseases due to fungi. Econazole is one of the broad-spectrum imidazole antifungal agents which are topically utilized to treat skin infections occurring due to numerous species of pathogenic fungi. Econazole is available as a cream or ointment commercially and is used for treating skin and other fungal infections. However, the salting effect of econazole cream leading to physical phase separation is the major problem encountered with this dosage form (cream) [18]. Corticosteroids are most commonly used to treat skin infections such as psoriasis, scleroderma, atopic dermatitis, etc. One of the well-tolerated corticosteroids used is triamcinolone acetonide. It exhibits low systemic toxicity when applied locally and is an effective anti-inflammatory agent. The exact mechanism on connective tissue is not completely known; however, it is expected that it diverts the synthesis of protein to carbohydrate formation, thus interfering with fibroplasia [19].

The objective of the current study was to develop co-encapsulated econazole–triamcinolone MSNs through the sol–gel method, then load these co-encapsulated MSNs into a gelatin scaffold to maintain sustained drug release locally over a prolonged time period for the clinical treatment of fungal skin diseases such as *Tinea pedis*, *Tinea cruris*, *Tinea corporis*, etc. This fabricated gelatin scaffold loaded with co-encapsulated econazole–triamcinolone MSNs was optimized using CCRD. Using a central composite rotatable design (CCRD), the procedure of scaffold development named as the freeze-gelation method was optimized. Statistical evaluation was used to assess the effects of the formulation variables on the entrapment effectiveness (EE) and drug release (DR) of EN-TA, and these variables included the concentration of gelatin (GT), the concentration of plasticizer, and the freezing time of the gelatin solution. To learn more about how the drugs interact with the carriers, the EN-TA-MSNs-loaded GT scaffold was subjected to analyses using X-ray diffraction, thermogravimetric analysis, and Fourier transform infrared spectroscopy (FT-IR). Then, the software-obtained optimized gelatin scaffold was subsequently tested for cytotoxicity, skin irritancy and in vitro, in vivo antifungal properties.

## 2. Results and Discussion

The entrapment of TA and EN in the developed nanoparticles ranged from 48 to 86% and 53 to 95%, respectively, as shown in Table 1. The entrapment of EN and TA in the scaffold formulation was found to be in between the ranges of 34–92% and 37–94%, respectively (Table 2). The formulation F4 of the scaffold exhibited a maximum entrapment efficiency of 92% for EN and 94% for TA, respectively, while the formulation F3 showed a minimum EE of 34% for EN and 37% for TA entrapment, respectively. It is quite evident that high concentrations of gelatin and plasticizer enhance the entrapment of EN and TA in the formulations up to a specific value, and this entrapment pattern was due to the change in integrity of the gelatin scaffold with changes in the concentrations of gelatin and plasticizer. As increased concentrations of gelatin and plasticizer lead towards more integrity and flexibility, so there was increased entrapment of the drug from the formulation; similarly, lower levels of gelatin and plasticizer lead to the decreased integrity and flexibility of the scaffold which resulted in the slower drug entrapment in the gelatin scaffold. The data for entrapment efficiency followed the quadratic model, and the software-generated polynomial equations are given below:

(1) $$EN\;Entrapment=69.78+3.68x_{1}+2.97x_{2}+0.6729x_{3}+1.12x_{1}x_{2}-1.37x_{1}x_{3}-0.3750x_{2}x_{3}-17.75x_{1}^{2}-24.43x_{2}^{2}+18.54x_{3}^{2}$$

(2) $$TA\;Entrapment=70.58+0.5510x_{1}+0.9010x_{2}+1.67x_{3}+2.63x_{1}x_{2}-0.6085x_{1}x_{3}-3.38x_{2}x_{3}-7.53x_{1}^{2}-29.37x_{2}^{2}+3.81x_{1}^{2}$$

It was clearly perceived from Equations (1) and (2) by polynomial values that *x*_1_(3.68) had a significant positive impact on the entrapment of both drugs because the coefficient value of *x*_1_ was found to be greater than the values of the other variables *x*_2_(2.97) and *x*_3_(0.673). Similarly, all variables contributed positively to improving the EE of EN and TA. The interactions *x*_1_*x*_2_(1.12) had synergistic interaction; however, *x*_1_*x*_3_(1.37) and *x*_2_*x*_3_(0.3750) were found to be antagonistic interactions for the EE of EN. For TA, a similar nature of the interaction of variables was observed. It was also observed that very high concentrations of gelatin (*x*_1_^2^) and plasticizer (*x*_2_^2^) exhibited an antagonistic impact on the entrapment of both drugs but a high level of freezing time (*x*_3_^2^) was found to be favorable for this purpose. The analysis of variance (Table 3) indicated that all studied formulation factors were found to be significant (<0.05) for the entrapment of EN and TA in the scaffold [20,21].

**Figure 1:**
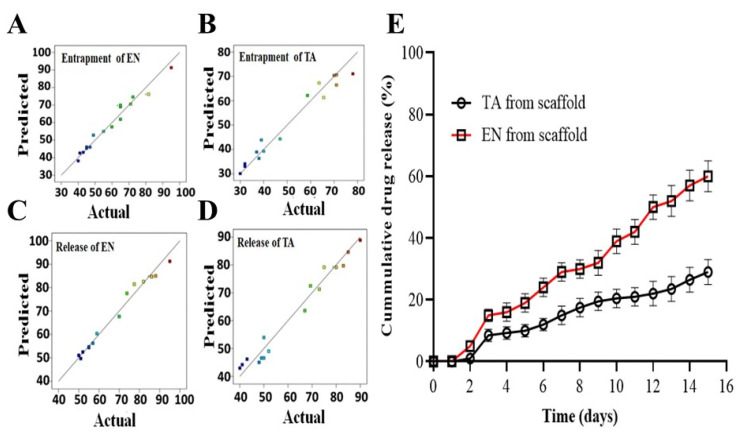
The graphs of predicted versus actual results of the entrapment of econazole (**A**), the entrapment of triamcinolone (**B**), the release of econazole from the scaffold (**C**), the release of triamcinolone from the scaffold (**D**) and the in vitro release profiles of econazole and triamcinolone from the optimized MSNs-loaded scaffold for 14 days (**E**). The colored dots in (**A**–**D**) indicated the predicted and actual value of EN and TA release from scaffolds.

The percentage release of EN and TA from the nanoparticles-loaded scaffold was found to be in between the ranges of 38–93% and 37–90%, respectively. The optimized formulation of the scaffold showing a percentage release of approximately 70% of EN and TA after 14 days of study is presented in Figure 1. The scaffold formulation F8 showed the highest release of econazole up to 93% and of triamcinolone up to 90%, which may be associated with lower levels of gelatin and plasticizer and a lower freezing time for the scaffold formulation, while the formulation F4 showed the slowest and least release of EN and TA (45%) because of the higher levels of the formulation variables used to formulate the scaffold.

High concentrations of gelatin and plasticizer prolonged the availability of TA and EN from the scaffold formulations up to a specific value and this release pattern was due to the change in strength and integrity of the gelatin scaffold. As an increased concentration of gelatin and plasticizer leads towards more compact integrity along with better flexibility, which caused a decrease in the percentage release of the drugs from the scaffold, similarly a low gelatin concentration and a low plasticizer level lead to the decreased integrity and flexibility of the scaffold, which results in a higher drug release from the gelatin scaffold. The CCRD-generated polynomial equations for the release of EN and TA from the nanoparticles-loaded scaffold are given below:

(3) $$EN\;Release=84.71+1.83x_{1}++0.5959x_{2}+0.5655x_{3}+2.13x_{1}x_{2}+1.39x_{1}x_{3}+0.1250x_{2}x_{3}+5.90x_{1}^{2}-31.09x_{2}^{2}+6.00x_{3}^{2}$$

(4) $$TA\;Release=79.17+2.12x_{1}+0.9283x_{2}+0.6227x_{3}+1.63x_{1}x_{2}+0.7025x_{1}x_{3}+1.12x_{2}x_{3}+15.79x_{1}^{2}-28.97x_{2}^{2}+12.40x_{3}^{2}$$

**Figure 2:**
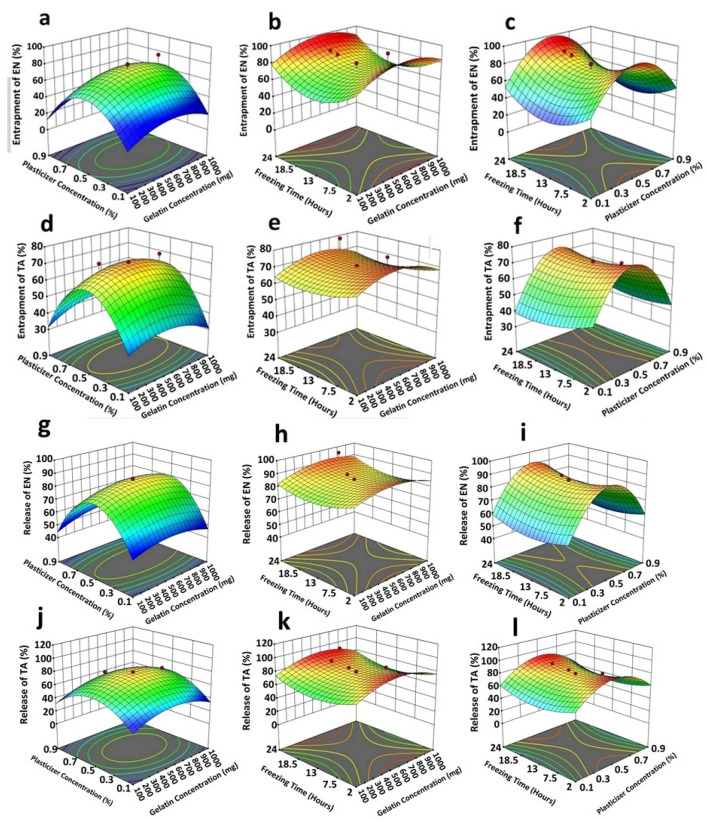
3D response surface plots indicating the following: the collective strong quadratic effect of plasticizer (*x*_1_) plus gelatin (*x*_2_) on entrapment efficiency of econazole (**a**); a medium quadratic influence of freezing time (*x*_3_) and gelatin (*x*_2_) on entrapment efficiency of econazole (**b**); a strong quadratic effect of plasticizer (*x*_1_) plus freezing time (*x*_3_) on entrapment efficiency of econazole (**c**); a strong quadratic effect of plasticizer (*x*_1_) plus gelatin (*x*_2_) on entrapment efficiency of triamcinolone (**d**); a weak quadratic influence of freezing time (*x*_3_) and gelatin (*x*_2_) on entrapment efficiency of triamcinolone (**e**); a strong quadratic effect of plasticizer (*x*_1_) plus freezing time (*x*_3_) on entrapment efficiency of triamcinolone (**f**); a strong quadratic influence of plasticizer (*x*_1_) plus gelatin (*x*_2_) in controlling econazole release (**g**); a very weak/negligible linear interactive influence of freezing time (*x*_3_) and gelatin (*x*_2_) in controlling econazole release (**h**); a strong quadratic effect of plasticizer (*x*_1_) plus freezing time (*x*_3_) in controlling econazole release from the scaffold (**i**); a weak quadratic effect of plasticizer (*x*_1_) plus gelatin (*x*_2_) in controlling triamcinolone release (**j**); a very weak/negligible linear interactive influence of freezing time (*x*_3_) and gelatin (*x*_2_) in controlling triamcinolone release (**k**);and a strong quadratic effect of plasticizer (*x*_1_) plus freezing time (*x*_3_) in controlling triamcinolone release (**l**) from the scaffold. The red dots and color of graphs showed the values of entrapment efficiency and release of EN and TA from prepared scaffolds.

The concentration of gelatin remained the most critical factor among the three for controlling and prolonging the drug release and, overall, all factors presented a synergistic impact. The interaction terms had also produced a synergistic effect on the drug release. This study demonstrated that a very high concentration of plasticizer (*x*_2_^2^) showed a prominent antagonizing impact on the drug release. Figure 1 also demonstrated that the obtained results were found to be closely in agreement with the predicted results suggested by the software for the four studied responses [21,22]. The results of ANOVA (Table 3) also showed the significant impact of all studied formulation factors (<0.05) regarding the release of both TA and EN from the nanoparticles-loaded scaffold. The cumulative impact of the formulation variables on the scaffold parameters is illustrated in the form of 3D graphs as given in Figure 2.

The correlation coefficients (R^2^) for the EE of EN and TA, and for the release of EN and TA, were observed to be 0.8852, 0.9210, 0.9665 and 0.9634, respectively. These obtained results were found to be very near to the results of the adjusted R^2^ results which clearly depict the adequacy of the applied model. The ratio of the maximum and the minimum values for the studied dependent variables (Y_1_ = 3.31, Y_2_ = 3.54, Y_3_ = 3.11, Y_4_ = 3.51) was observed to be less than three, suggesting that there was no need to change the model, and the *p*-value was <0.05 indicating that the quadratic applied model was significant. The data of all studied responses displayed a good fit with the quadratic model. The adequacy and suitability of the quadratic model applied for the dependent variables was further demonstrated from the signal-to-noise ratio calculated from the adequate precision, which should be >4, and its results were found to be 19.862 for Y_1_, 18.762 for Y_2_, 16.482 for Y_3_ and 14.48 for Y_4_ (Table 4).

For further validation and optimization of the scaffold formulation, a batch of optimized scaffold formulations was synthesized by applying the suggested optimized levels of the formulation variables (Table 5) as recommended from the software Design Expert (version 8.0.6.1 Stat-ease, Inc., Minneapolis, MN, USA).

The formulation of the optimized scaffold batch was established to attain a higher EE (90%) and a highly controlled release of drugs (40%) by using the numerical optimization procedure. According to the desirability factor, the software-suggested levels of the formulation variables and desirable results were prioritized, and the desirability factor was calculated to be near one for all studied responses. The software-suggested and -generated optimized batch of scaffolds was formulated having a zeta potential of −16 mV (Table 5) and was further subjected to be analyzed for various biomedical uses. The optimized batch of formulated scaffolds showed an EE of 94% for both drugs and a 48% and 35% release of TA and EN, respectively. The gelatin scaffold loaded with MSNs having cross-linked nanoparticles showed a 89% and 92% EE of TA and EN, respectively, and the releases of EN and TA were 31% and 41%, respectively.

**Figure 3:**
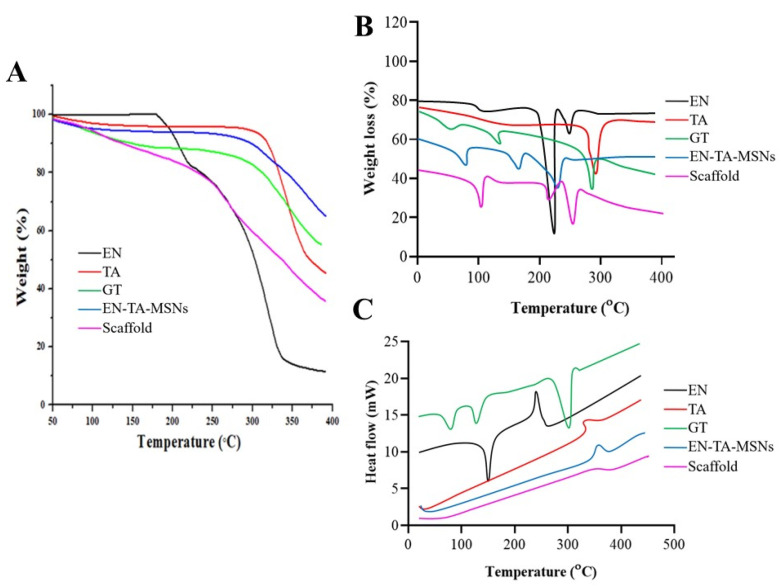
TGA (**A**), dTG (**B**) and DSC (**C**) thermograms of EN (econazole nitrate), TA (triamcinolone acetonide), GT (pure gelatin), EN-TA-MSNs and SC (scaffold).

The entrapment efficiency and the drug-loading analyses were supplemented by Thermogravimetric Analysis (TGA) as a function of weight loss with a rise in temperature. The TGA/dTG curves of EN and TA demonstrated a weight loss at two different points, as depicted in Figure 3A. The first weight loss was most probably due to loss of humidity, while the greater weight loss was related to the degradation of the drugs. The thermal analysis of the pure gelatin and the drug-loaded MSNs and the drug-loaded MSNs encapsulated in the gelatin scaffold revealed the loss of weight at three different points. In the case of the gelatin, the initial weight loss was due to the loss of moisture; the second and third weight losses were due to the degradations of the protein gelatin. For the EN-TA-loaded MSNs, the initial weight loss (4.66%) was found at 94.27 °C which was probably due to the removal of moisture. The second but more minor weight loss (4.04%) at 288.12 °C could be associated with the breakdown of organic matter, while another major weight loss (23.36%) was noted at 381.01 °C which could be associated with the degradation of the drugs. While in the case of the drugs-loaded gelatin scaffold, the loss of weight was depicted at three points at 100.91 °C, 251.15 °C and 372.48 °C, respectively. The first weight loss (5.83%) was due to the removal of moisture and superfluous impurities leading towards the cracking in the scaffold structure as depicted from the second weight loss (18.28%), and another significant loss of weight (36.22%) was certainly linked with the breakdown and degradation of the whole scaffold formulation. Losses of weight of 32.02% and 60.33% were observed for the EN-TA-MSNs and for the EN-TA-MSNs-GT scaffold, respectively, which were most probably due to drug uptake. The maximum encapsulation of both drugs in the scaffold matrix was quite evident from the greater weight loss from the scaffold formulation. The entrapped amounts of drugs as recovered in the HPLC analysis and presented in Table 2 were observed to be quite in agreement with those values obtained by TGA [23,24]. These outcomes may also be associated withthe SEM observations that the gelatin scaffold has a highly porous structure which is considered as most advantageous to entrap drugs into its pores.

DSC thermograms of pure drugs, drugs containing MSNs and MSNs-loaded GT scaffolds are presented in Figure 3B. The DSC of EN and TA revealed peaks close to their melting points indicating the crystalline nature of both of the drugs. However, the DSC analysis of the gelatin exposed three peaks. Three endothermic peaks were observed at 91 °C, 113 °C and 301 °C due to the decomposition of the gelatin [25,26]. The endothermic peak observed at 91 °C was linked to the removal of residual water. The DSC analysis of the drugs-loaded MSNs encapsulated in the gelatin scaffold EN-TA-MSNs-GT exhibited water desorption phenomena, while the peaks relevant to the melting points of EN and TA were not detected, in agreement with the existence of an amorphous form of the drugs inside the pore structure of the scaffold as revealed by the XRD analysis. No remarkable difference in the DSC peaks of the gelatin and scaffold was observed. This is evidence of the phase transition of all the components of the formulations to amorphous forms and their homogenous distribution in the scaffold [27].

**Figure 4:**
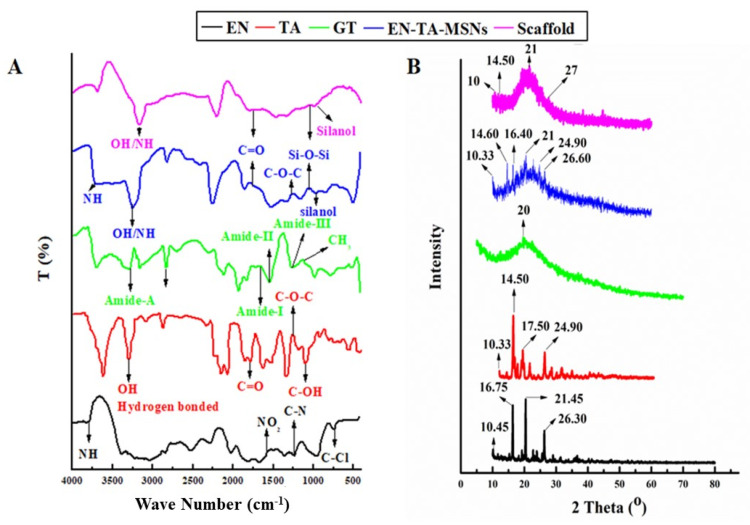
FTIR (**A**) and XRD (**B**) spectra for EN (econazole nitrate), TA (triamcinolone acetonide), GT (pure gelatin) and SC (scaffold).

FTIR studies were carried out to ascertain the probability of any possible interaction between the drugs, gelatin, silica nanoparticles and scaffold (Figure 4). The FTIR analysis of pure EN exhibited an absorption pattern at wave number 3811 cm^−1^ indicating the -NH group’s stretching frequency. The stretching frequency observed at 1568 cm^−1^ indicated the occurrence of the NO_2_ functional group. The absorption exhibited at wave number 1220 cm^−1^ was linked to the presence of the C-N group.

The pure drug TA showed a typical absorption infrared band at the wave number 3396.64 cm^−1^ which can be linked to the existence of the hydrogen-bonded hydroxyl group. The absorption band at 1774 cm^−1^ was linked to the stretching frequency of the carbonyl (C=O) group. The absorption band at the wave number 1270 cm^−1^ displayed the C-O-C bond in the aliphatic esters. The absorption frequency at 1062.78 cm^−1^ indicated the C-OH (aromatic) bond. The FTIR spectra of drugs-loaded nanoparticles exhibited the characteristic peaks of Si-O-Si at 1050 cm^−1^ and silanol (-OH) at peaks 940 cm^−1^ as presented in Figure 4A. The finally formulated MSNs loaded with drugs have not displayed any new peak related to the structural interaction between the drugs and silica. However, minor variance in the absorption frequencies and in the intensity of the peaks of the drugs was observed [28]. The FTIR analysis for pure gelatin exhibited the characteristic peak at 3433 cm^−1^ attributed to the-NH bond of amide-A. The presence of amide-I was also depicted from the absorption band at wave number 1630 cm^−1^. Another peak at 1565 cm^−1^ revealed amide-II and a band observed at 1240 revealed amide-III. The asymmetric and symmetric bending vibrations of the methyl group were visualized from the absorption frequency that appeared between wave number 1460 cm^−1^ and 1380 cm^−1^ [23]. The FTIR analysis results of the drug-loaded MSNs encapsulated in the gelatin scaffold confirmed all the major peaks (with slight shifting) of the pure gelatin, EN and TA, thus confirming the fact of the lack of any significant structural interaction of the drugs to the formulation ingredients.

X-ray diffraction analysis was employed to determine the phase transition of the drugs after entrapment into the formulation. The XRD of pure EN, TA, drugs-loaded MSNs and scaffolds are presented in Figure 4B. The results depicted that the drugs retained their original crystallinity inside the MSNs but transformed into an amorphous form in the gelatin scaffold. The XRD of the pure drug EN exhibited three major peaks at 16°, 20° and 26° which made evident its crystalline nature [29]. Similarly, the XRD of TA exhibited four major peaks at 15°, 17°, 20° and 25° which revealed its crystalline structure [30]. A pronounced peak found at 20° in the XRD pattern of pure gelatin revealed that its structure was essentially amorphous in nature. There were minor depressions in the intensity of the peaks of the drugs in the diffractogram of the drugs-loaded MSNs. However, substantial fading of the intensity of the drug peaks was detected in the XRD pattern of the EN-TA-loaded MSNs encapsulated in the gelatin scaffold. Changes in the diffractogram revealed the entrapment of the drugs into the silica nanoparticles and then their encapsulation into the gelatin scaffold leading towards the amorphous physical state of the encapsulated drugs [31].

**Figure 5:**
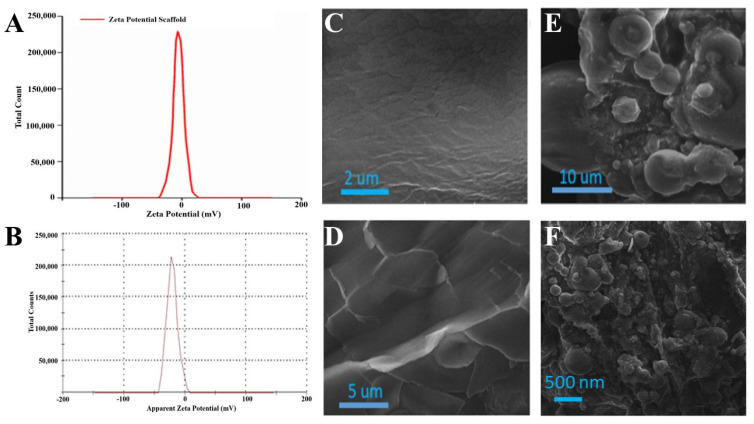
Zeta potential analysis of MSNs-loaded scaffold (**A**), zeta potential of EN-TA-loaded scaffolds (**B**), SEM micrographs of blank gelatin (**C**,**D**), MSNs-loaded scaffold (**E**) and SEM image of EN-TA-loaded scaffolds (**F**).

Zeta potential is a charge difference in between a stationary layer, within which particles are dispersed, and two dispersion media. Zeta potential may be positive or negative depending on the chemistry and polarity of the particles. The zeta potential of the EN-TA-loaded gelatin scaffold was performed by a zeta potential analyzer, as shown in Figure 5A. Only a single peak was observed at −16.20 which covered 100% of the area. This negative potential was attributed to the negatively charged hydroxyl groups (-OH) present in the silica nanoparticles loaded into the scaffold [32]. The poly dispersity index value of the silica nanoparticles was found to be 0.49, which demonstrated the fairly uniform size distribution of the prepared nanoparticles. This size distribution also verified the stability of the drugs-loaded MSNs, which was an essential parameter for ensuring the suitability of MSNs administration. The value of the zeta potential of the EN-TA-loaded MSNs was in having a single net peak at −23 mV totally covering 100% of the area. Therefore, the MSNs containing the co-encapsulated EN-TA were considered highly stable as there was a complete absence of the aggregation of nanoparticles because the results of the zeta potential were within the well-defined limits. A nano-system is considered stable when all of the particles are either negatively or positively charged; otherwise the particles having opposite charges will attract each other and will be considered less stable.

SEM is an expedient tool to determine surface morphology. The SEM micrographs of blank gelatin (GT) and the scaffold (EN-TA-MSNs-GT) are presented in Figure 5. The SEM analysis of blank gelatin was found heterogeneous with a rough and uneven surface (Figure 5C,D). In contrast, the fabricated scaffold had a highly porous structure (Figure 5E). The porous structure of the scaffold is necessary for the better permeability of drugs and favors the supply of oxygen. All EN-TA-loaded MSNs were exclusively observed to be uniformly monodispersed, spherical in shape with about a less than 500 nm average size, as mentioned in Figure 5F [33].

**Figure 6:**
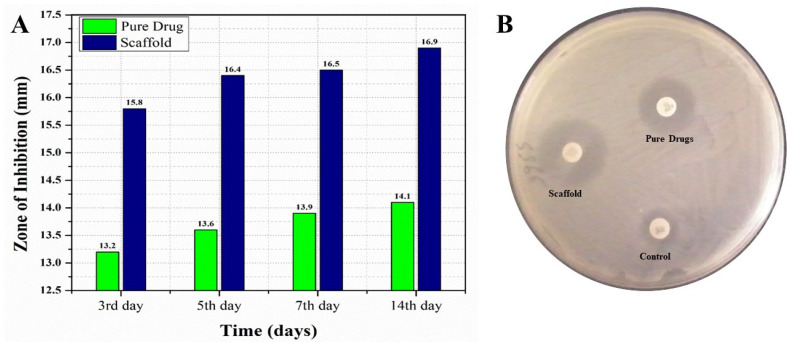
The comparative in vitro antifungal evaluations of the pure drugs and MSNs-loaded scaffold with their respective zones of inhibition at day 3, day 5, day 7 and day 14 (**A**) and the zones of inhibition of the control, the pure drugs and the scaffold (**B**).

For conducting a comparative in vitro antifungal analysis of the pure EN-TA and gelatin scaffold, all the formulations were tested at day 3, 5, 7 and 14 of study, and zones of inhibition were obtained by these formulations. Differences were found in between the zones of inhibition of these two tested formulations. On the third day of study, a zone of inhibition of 13.20 mm was demonstrated by the suspension of pure drugs and a zone of 15.80 mm was produced by the MSNs-loaded gelatin scaffold, as shown in Figure 6. At the fifth day, the zone of inhibition from the suspension of pure drugs was 13.60 mm and the zone was 16.40 mm from the MSNs-loaded gelatin scaffold, as shown in Figure 6. At the seventh day, the zone of inhibition produced by the pure drugs suspension was 13.90 mm and a 16.50 mm zone was produced by the MSNs-loaded gelatin scaffold, as shown in Figure 6. At the fourteenth day, an inhibition zone of 14.10 mm was observed for the suspension of pure drugs and a zone of 16.90 mm was demonstrated by the MSNs-loaded gelatin scaffold. The findings of this study clearly prove that the antifungal efficacy of the gelation scaffold was best with the greatest zone of inhibition measured, which made the scaffold superior with greater acceptability as compared to the pure drugs suspension [34,35].

In this study, a beneficial effect of the gelatin in the scaffold containing the drug-loaded nanoparticles on antifungal activity was observed. Such a positive impact of the gelatin for a better and improved antifungal activity [36] was also observed by Amit et al. in designing hydrogels loaded with peptides with a gelatin which delivered a prolonged release of the drug up to 24 h. The miconazole nitrate-loaded films formulated from different polymers, such as gelatin, alginate, arabic gum, carbopol and chitosan, by Tejada et al. also showed similar effectiveness [37]. In another study, a gelatin capsule (GEL-CuNPs) containing copper nanoparticles (CuNPs) also demonstrated the positive antifungal activity of gelatin [38]. In contrast to drugs suspension where the whole amounts of the drugs are available at once, the gelatin scaffold provided a prolonged and sustained release of the drugs because of the improved swelling capacity and the muco-adhesiveness of gelatin. Furthermore, the presence of a specific sequence of amino acids such as Arg-Gly-Asp present in gelatin may be considered as responsible for better cell adhesion and drug delivery [39].

**Figure 7:**
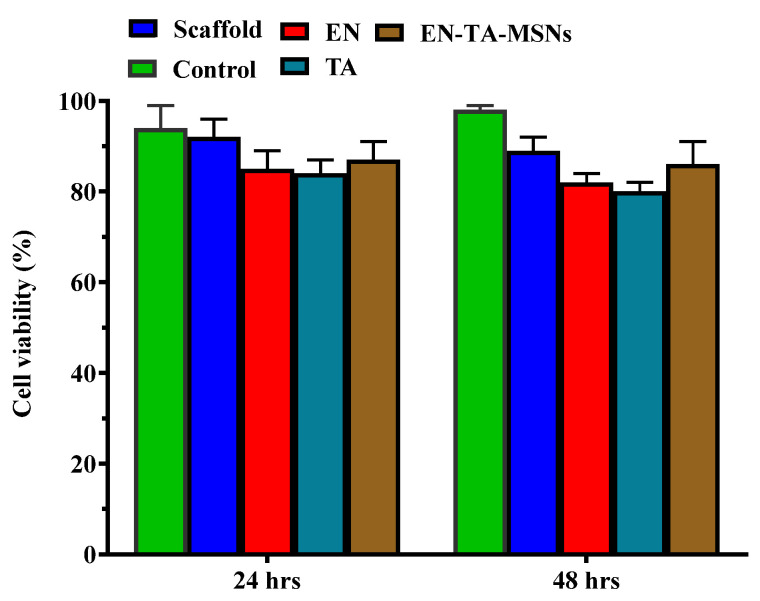
Cytotoxicity evaluation of pure econazole, pure triamcinolone, gelatin and MSNs-loaded scaffold for 6 and 24 h.

Cell survival was found to be about 89% and 86% with the scaffold and the EN-TA-MSNs, respectively. Even after 48 h, the cell viability of the scaffold and the EN-TA-MSNs was more than 85%, suggesting no cytotoxic effect of the EN-TA-MSNs, as shown in Figure 7. The outcomes of current cytotoxic studies exposed that the cell survival percentage for the scaffold-treated group was much more than that of the pure drug-treated group, depicting the comparatively reduced cytotoxic potential and the enhanced safety character of the scaffold as compared to the studied pure drugs [40,41].

**Figure 8:**
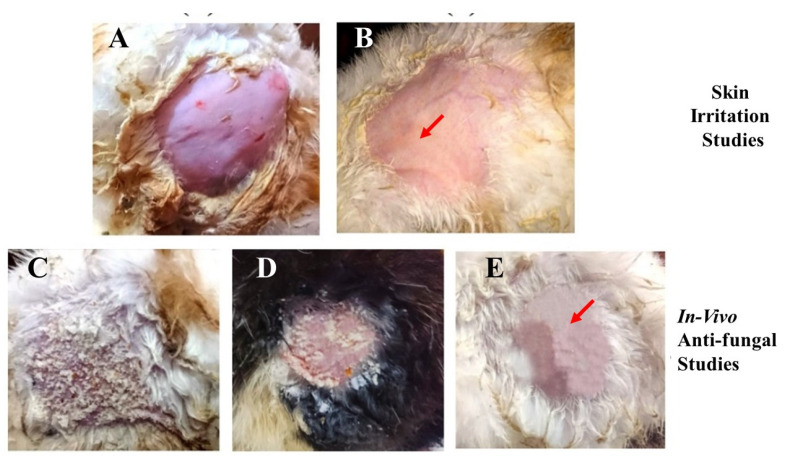
Skin irritation studies in (**A**) control group; (**B**) group receiving treatment with the MSNs-drug-loaded scaffold; the in vivo antifungal activity of the control group (**C**); the pure drug-treated group (**D**); and the drug-loaded MSNs in the gelatin scaffold (**E**).

The conventional use of EN-TA for treatment contains drawbacks such as skin irritation, inflammation and erythema which powerfully hamper the tolerability and applications of such treatment by patients. Preferably, the drug-delivery mode of econazole–triamcinolone should be able to lessen or eliminate these erythematic incidents. The basic assumption was that the encapsulation of EN-TA drugs into the MSNs and then loading the MSNs into a scaffold would decrease the persistent interaction of both drugs with the skin stratum corneum (and so limit the activating feature that initiates the erythema episodes) and, consequently, result in fewer erythematic events. The outcomes acquired after the primary skin irritation trials are elaborated in Table 6. The 14-day skin irritation trials specified that the enrichment of the scaffold with the co-drugs-loaded MSNs was proven to be significantly less irritating as compared to the suspension of the pure drugs, as shown in Figure 8.

A continuous increase in skin irritation or erythema was observed after several days of treatment in the group of animals treated with the suspension of pure drugs; however, no erythema sign was spotted in the group treated with the MSNs-loaded scaffold. Consequently, the MSNs-based scaffold demonstrated a superior action over the pure drugs suspension and thus confirmed the improved tolerability of the drugs towards skin. This advantage might be useful in refining patients’ compliance for the topical delivery of EN-TA [36]. The in vivo antifungal efficacy of the MSNs-loaded scaffold was performed on rabbits. Twenty-four female and male rabbits (1.5–2 kg) were taken and divided into three groups, each group comprising six animals. Isolated colonies of *Candida albicans* were utilized for the induction of cutaneous candidiasis in the rabbits.

Table 6 represents the comparative effectiveness of the econazole–triamcinolone suspension and the MSNs-loaded gelatin scaffold in animals against cutaneous candidiasis. From the lacerations of the treated animals, the colonies of viable *Candida albicans* were isolated and detached. In groups treated with the suspension of drugs, four rabbits out of six demonstrated positive culture tests. The superior efficacy in the treatment of candidiasis was clearly verified from animals treated with the MSNs-loaded scaffold because a positive culture test was not demonstrated even from a single rabbit, as shown in Figure 8. However, the control group, in which no treatment was given to any rabbits, showed positive culture tests. From the fungal infection, the rapid recovery was also quite evident in the group treated with the MSNs-loaded scaffold because of the lack of any sign of a positive culture of *Candida albicans*. This remarkable efficacy of the MSNs-loaded gelatin scaffold in treating fungal infections can be associated with better bio-adhesiveness, a highly occlusive nature and a good controlled release of drugs for a prolonged time [42]. Accelerated stability studies of the gelatin scaffold were performed for three months and their physical texture/integrity along with an assessment of the drug contents were found to be within defined limits. No brittleness or trimming of the scaffold parts were detected. The drug contents were also found to be within a 95–97% level.

## 3. Materials and Methods

### 3.1. Materials

Triamcinolone acetonide (TA) and econazole nitrate (EN) were acquired as gift samples from Mass Pharma, Lahore, Pakistan, and from Harman Pharmaceutical, Lahore, Pakistan, respectively. Ammonium hydroxide, gelatin, hydrochloric acid, sorbitol, glycerol and absolute ethanol were purchased from Sigma Aldrich, Darmstadt, Germany. Vegetable oil was purchased from Dalda Foods Pvt. Ltd., Nishat commercial area, Itehad colony, Karachi, Pakistan. Ethyl silicate was purchased from Uni-Chem, Mumbai, India.

### 3.2. Design of Experimental Work

The Design Expert Software was used for the generation and optimization of the formulation variables of the MSNs-loaded gelatin scaffold. The methodology of central composite rotatable design (CCRD) was applied for the investigation of the individual and combined effects of the selected variables on the responses. The entrapment of EN (Y_1_), entrapment of TA (Y_2_), release of EN (Y_3_) and release of TA (Y_4_) were taken as responses and categorized as dependent variables. The concentration of gelatin (*x*_1_), the concentration of plasticizer (*x*_2_) and the freezing time of the scaffold (*x*_3_) were categorized as independent variables. The results were statistically optimized by using the analyses of variance ANOVA, regression analysis and lack-of-fit tests. By comparing the PRESS value of all suggested models, the best-fitted model with the lowest PRESS was selected [20].

### 3.3. Preparation of Co-Drugs-Loaded Mesoporous Silica Nanoparticles

The preparation of MSNs loaded with both drugs was carried out by sol–gel methodology. In this process, distilled water and freshly made 0.1 M HCl were slowly added to ethyl silicate, and the mixture was thoroughly agitated for five minutes at a speed of 200 rpm on the magnetic stirrer. Solutions of EN (10%) and TA (3%) separately made in ethanol were added to this sol. The drugs containing sol were cooled to 4 °C and pH at 5.8 was maintained by 0.08 M of NH_4_OH, and gelling time was noted [43]. The resulting gel was then incrementally added to vegetable oil (100 mL) while being continuously stirred at 1000 rpm with a yellow line homogenizer until nanoparticles began to form. Filtration was used to isolate the silica nanoparticles, which were then cleaned with distilled water and allowed to air-dry.

### 3.4. Preparation of MSNs-Loaded Gelatin Scaffold

To prepare the scaffold, 800 mg of gelatin powder was completely dissolved in 9 mL deionized water at 40 °C. To this solution, 1 mL of the parent solution (EN-TA-loaded MSNs), 2–3 drops of glycerin and 3 mg of sorbitol were added. The resulting mixture was vigorously mixed to obtain a homogeneous solution. Afterwards, the solution was carefully injected into the mold without bubbles inside. The gel was developed after soaking at a temperature of 4 °C for 1 h, followed by a decrease in temperature to −20 and −80 °C. When the gel was completely frozen, the gelatin matrix freeze-dried to form a scaffold [44]. The Design Expert Software was used to design the composition of different scaffold formulations according to Table 1 and twenty different formulations were prepared before starting the optimization process. The MSNs of the optimized formulation were also prepared using glutaraldehyde as a cross-linker and these nanoparticles were loaded into the gelation scaffold.

### 3.5. Evaluation of MSNs-Loaded Scaffold

#### 3.5.1. Entrapment Efficiency of Drugs in Nanoparticles and MSNs-Loaded Scaffold

The developed nanoparticles and a piece of scaffold about one inch in length were transferred to 25 mL of phosphate buffer solution having a pH of 7.4 and stirred slowly for 24 h. It was further sonicated to assure that the whole amount of loaded drugs was released from the scaffold. After that, the mixture was filtered and dilutions were prepared for the standard and working solutions [21,40]. Then, using a previously established HPLC procedure, the solutions were examined to determine the amount of medication present. Equation (5) was used to calculate the amount of each drug entrapped in each scaffold formulation.

(5) $$Entrapmentefficiency=\frac{estimateddrugcontent}{theoreticaldrugcontent}\times 100$$

#### 3.5.2. Drug-Release Studies from MSNs-Loaded Scaffold

The in vitro release of EN and TA from the MSNs-loaded scaffold was carried out by using the Franz diffusion cell method [45]. The drug-release process was performed in freshly prepared phosphate buffer at pH 7.4 and 37 °C. Cellulose acetate membranes (Sartorius^®^, Darmstadt, Germany, 0.45 μm average pore size) were soaked in distilled water for 30 min before loading the SC formulation and then fixed on top of the receptor chambers of the cells after adding the release media in the chamber. The donor compartment was tightly clamped on the membrane fixed on top of the receiver compartment. About 150 mg of the SC formulation was loaded in the donor chamber already containing 10 mL of the release medium at a stirring speed of 50 rpm. At predetermined time intervals, about 0.5 mL of the sample from the receptor chambers of the Franz diffusion cell were taken and 0.5 mL of freshly prepared media was added in that chamber. After the appropriate dilution, the amount of EN and TA in the obtained samples was determined using a previously determined HPLC technique.

#### 3.5.3. Thermal Analysis

The TGA/DSC-1 system (Mettler-Toledo, Im Langacher 44, 8606, Greifensee, Switzerland) was used to perform the thermal characterization of the TA, EN, gelatin and MSNs-loaded gelatin scaffold. The samples were exposed to the instrument after being sealed inside an aluminum pan. Heat was applied at a rate of 20 °C per minute at temperatures between 40 and 300 °C. To maintain the atmosphere’s inertness, nitrogen was pumped at a rate of 50 mL/min [23].

#### 3.5.4. Fourier Transform Infrared (FTIR) Spectroscopy

The FTIR spectra were recorded by using an IR instrument, (Shimadzu—prestige P21). The scanning range of 400–4000 cm^−1^ was adopted for 20 scans at a resolution of 4 cm^−1^.

#### 3.5.5. Crystallographic Evaluation by X-ray Diffractometer (XRD)

The scaffold was subjected to an X-ray diffraction study utilizing the D8 advance X-ray (Bruker AXS, Madison, WI, USA) at 40 kV voltage using 40 mA current. The treatment of samples with monochromatic graphite and copper material was performed at an adjusted voltage of 40 kV [46].

#### 3.5.6. Scanning Electron Microscope (SEM) Analysis

Scanning electron microscope analysis was carried out for surface morphology determination of the blank scaffold and the MSNs-loaded gelatin scaffold with MIRA 3 TESCAN Huntsville, AL, USA. The samples were gold-coated and positioned on one side of the double adhesive stub and then examined.

#### 3.5.7. Analysis of Particle Size and Charge of MSNs-Loaded Scaffold

The analysis of the zeta potential of the silica nanoparticles-loaded gelatin scaffold was determined by a zeta sizer (Malvern version 7.11). The samples were positioned into an electrophoretic cell with an automatic mode to measure charges on the scaffold and SC at 25 °C [28]. The determination of the poly dispersity index of the scaffold was conducted by using the following formula, where ‘Mn’ and ‘Mw’ were average number and average weight, respectively.

(6) Polydispersity index=Mw/Mn

### 3.6. Biomedical Evaluation of Optimized Scaffold

#### 3.6.1. Skin Irritation Testing

Draize patch tests were used to compare the potential for two medicines to irritate the skin when they were administered as simple suspension-form drugs, drugs-loaded MSNs and MSNs-loaded gelatin scaffolds to the rabbit models [47]. An endorsement of the designed study was obtained from the Ethical Committee of the College of Pharmacy, University of Sargodha, Pakistan (EC N0: 106 May, 20 for six month). Three groups of rabbits, each having three rabbits, were made. Group I (control group) did not receive any treatment while group II and group III were treated with the suspension of pure drugs and the MSNs-loaded gelatin scaffold, respectively. The rabbits’ back hair was shaved 24 h before the application of the MSNs-loaded scaffold and the EN-TA suspension. The tested formulations were evenly applied to the 3 cm^2^ surface area of hairless skin. The skin was then checked for observable changes, such as erythema, on the first day, third day, seventh day and fourteenth day after the application of the formulations. The mean erythema values, which depend on the erythema measurement and range from 0 to 4, were recorded.

The scale of erythema was from 0 (no erythema sign), 1 (minor erythema showing light pink color), 2 (modest erythema with dark pink color), 3 (slightly severe erythema with light red color) to 4 (severe erythema with intense redness).

#### 3.6.2. In Vitro Antifungal Studies

For in vitro antifungal evaluation, various formulations, i.e., pure EN-TA and gelatin scaffold loaded with MSNs, were suspended into agarose medium at 37 °C. *Candida albicans* strain was utilized in the study and the diffusion test was applied on Mueller–Hinton Agar according to the process specified by the National Committee for clinical laboratories after a slight modification [48]. Micro-organisms were maintained at 37 ± 1 °C aerobically. The strains of *Candida albicans* were obtained by transferring a colony of *C. albicans* to Sabouraud dextrose broth from agar plates by the procedure of inoculation and then it was maintained for the whole night in aerobic conditions at 35 °C. Then culture was centrifuged for 10 min at 1000 rpm, after which the cells were again suspended into 0.9% NaCl for matching the turbidity standard with 0.5 McFarland turbidity standards. The surface of the Mueller–Hinton and 3% agar glucose plates were infected with test strains of *Candida albicans* after the turbidity was adjusted within 15 min. The agar plates were inoculated using a sterile cotton swab by streaking on the whole surface of the agar, and streaking was repeated twice by simply turning the agar plate at a temperature of about 60 °C. Then, a well cavity (3–4 mm) was punched, and each cavity of the agar plates received separate additions of 50 µL of pure EN-TA suspension prepared by using 5% carboxymethyl cellulose in water and 50 µL of the scaffold of EN-TA-loaded MSNs in water. These two suspensions contained 35 µM of TA and 40 µM of EN. Agar plates were brought to room temperature for two to three minutes before being incubated at 35 °C for 24 h under aerobic conditions. By employing calipers, the zone of inhibition was determined from a sharp drop in growth density.

#### 3.6.3. Cytotoxicity Studies

Human skin dermal fibroblast cells (HDFa) (3 × 10^3^ cells/well) were cultured in a 96-well plate with full Dulbecco’s modified Eagle’s medium (DMEM) containing 10% fetal bovine serum (FBS), 100 U/mL penicillin and 100 μg/mL streptomycin, and incubated at 37 °C with 5% CO_2_ for 24 h. Just a day before initiating the studies, DMEM without FBS was used to feed the cells. The combined pure drugs TA (35 µM) and EN (40 µM) as well as the individual pure drugs and the scaffold and the EN-TA-MSNs were dispersed in DMEM and incubated with HDFa cells for 24 h and 48 h to evaluate the potential cytotoxic effects while maintaining Triton X-100 and pure DMEM as negative and positive controls, respectively. At the end of the study time, a 5 mg/mL solution of MTT in PBS (20 μL) was poured in each well followed by incubation at 37 °C for 3–4 h. After completion of incubation time, the MTT solution was removed and dimethyl sulfoxide (100 μL) was supplemented in each well. The reaction of MTT with mitochondrial reductase enzyme caused the production of formazan crystals which were extracted from the cells by rotating the mixture in a shaker for 15 min. The analysis of the plates was performed at 540 nm in an ELISA reader (Bio Rad, Hercules, CA, USA) and the rate of cell viability was calculated against considering the 100% cell viability of cells treated with DMEM [49]. Cell viability rates were calculated according to the following equation:

(7) $$Cell\;viability\left(\%\right)=\frac{A_{S}}{A_{d}}\times 100$$

The absorbance measured after the treatment with the sample dispersions was denoted as “A_S_” and the absorbance measured after treatment with DMEM was noted as “A_d_”.

#### 3.6.4. In Vivo Antifungal Studies

For evaluation of the in vivo antifungal efficacy of the prepared drug formulations, accurately weighed rabbits (1.5–2 kg) were carefully selected and then categorized into three groups in such a way that six rabbits were present in each group. To induce fungal infection, *Candida albicans* (MTCC 183) was utilized. The fungal infection candidiasis was created on the cutaneous tissue of the rabbits by following the previously described method with a small alteration [40]. Simply, the hairs from the back of the rabbits at an area of 4× 4 cm^2^ were removed by the use of dermatologically proven hair-removing cream. The skin area was scratched slightly on the next day by using sandpaper followed by the application of an already-prepared inoculum (600 mg) of *Candida albicans* with the help of a glass rod. Various formulations, i.e., the pure EN and TA suspensions and the MSNs-loaded scaffold, were then used against candidiasis for fourteen days, beginning from the post-infection day, on the rabbits of the two treated groups, exclusive of the rabbits of the control group. The rabbits of the control group were ensured to be infected but no formulation for treatment purposes was administered to them. Just after the commencement of therapy, the animals were visually observed thoroughly to detect any changes in the texture of the skin at the site of infection. The skin texture of the rabbits was analyzed in order to note the time required for treatment in each group and the antifungal efficacy of all studied formulations for treating mycosis was then compared with the control group. Studies of the culture were performed to evaluate the effectiveness of therapy. At the completion of each therapy (14 days), a small skin segment from the area of treatment was removed. The segment was subjected to homogenization with saline with the help of a tissue homogenizer, and a small percentage of the obtained homogenate was then spread on the solidified growth medium, i.e., the agar chrome. The incubation of the prepared Petri plates was performed at 25 °C in the incubator for 6 days. The number of colony-forming units (CFUs) in each of the chrome agar plates was counted, and then for each infected group, the logarithm of the number of CFUs was calculated. The studied rabbits were given a status of positive for fungal infection after the observation of more than one colony.

#### 3.6.5. Stability Studies

Utilizing ICH recommendations, accelerated stability investigations were performed at a temperature of 40 ± 2 °C and a relative humidity of 75 ± 5%. The scaffold’s physical characteristics and drug contents were evaluated.

## 4. Conclusions

A well-designed, optimized scaffold was prepared for the prolonged local delivery of drugs with an aim to sustain and extemporize the synergistic action of the drugs, thereby addressing the glitches such as the requirement of higher drug concentrations and repetitive uses, the irritating and inflammatory impacts on skin and the poor drug penetration through the dermis. Regarding the stated objectives, the econazole and triamcinolone co-loaded mesoporous silica nanoparticles (MSNs) were loaded into a gelatin scaffold and optimized through CCRD to achieve the best possible sustained and local drug release. The MSNs-loaded scaffolds were then developed by a very convenient, simple and expedient technique for the simultaneous and prolonged delivery of co-encapsulated antifungal agents for an effective cure of the most resistant and common fungal infections. The experimental parameters were successfully optimized for not only having increased EE but also for having a controlled and prolonged drug release at the site of action. The in vitro evaluations such as the TGA/DSC, XRD and FTIR analyses indicated an excellent compatibility of both drugs with the formulation ingredients. The SEM studies demonstrated the porous structure of the scaffold containing uniformly distributed co-drugs-loaded MSNs within the scaffold. Comparatively, the scaffold enriched with econazole–triamcinolone-loaded MSNs proved to be less cytotoxic as compared to the pure drugs, which might be due to the higher occlusive and bio-adhesive characteristics of the gelatin scaffold specifically designed for the prolonged controlled delivery of both drugs. The MSNs-loaded scaffold will definitely increase the adherence and tolerance in patients by overcoming the drawbacks such as rashes, itching and dryness of skin mostly associated with the traditional methods of use of the two drugs. The toxicity of the co-encapsulated antifungal drugs was significantly decreased by the design of the combination-therapy-based optimized scaffold formulations. The most important aspect of this study is the achievement of the synergistic antifungal efficacy of the combination therapy in both in vitro and in vivo environments at the reduced dose of both drugs loaded in the MSNs. The favorable outcomes from a wide range of in vitro and in vivo testing prove the gelatin scaffold to have super-efficient and appropriate controlled drug release and antifungal efficacy for the resistant fungal infections.

## Figures and Tables

**Table 1:** Percent entrapment efficiency (%EE) of TA- and EN-loaded silica nanoparticles.

Formulation	%EE of TA	%EE of EN	Formulation	%EE of TA	%EE of EN
F1	82 ± 2.63	95 ± 3.92	F11	53 ± 2.83	55 ± 3.34
F2	71 ± 1.95	73 ± 2.81	F12	86 ± 3.42	90 ± 2.87
F3	86 ± 2.31	87 ± 1.71	F13	50 ± 3.57	53 ± 2.58
F4	66 ± 1.88	68 ± 2.62	F14	52 ± 2.37	54 ± 2.69
F5	81 ± 3.36	84 ± 3.96	F15	84 ± 2.85	89 ± 3.89
F6	72 ± 3.82	73 ± 3.73	F16	67 ± 3.83	72 ± 2.74
F7	75 ± 2.59	77 ± 2.98	F17	48 ± 1.38	51 ± 3.46
F8	73 ± 3.78	75 ± 2.93	F18	49 ± 2.82	53 ± 2.27
F9	72 ± 2.28	73 ± 2.86	F19	72 ± 1.94	75 ± 3.19
F10	69 ± 1.37	72 ± 2.38	F20	85 ± 2.42	91 ± 3.85

**Table 2:** Composition of different scaffold formulations as suggested by Central Composite Rotatable Design and the results of entrapment efficiency and release of econazole and triamcinolone.

Formulation	Formulation Variables				Results of Responses		
	Gelatin Conc. (mg/100 mL)	Plasticizer Conc. (mg/100 mL)	Freezing Time	Entrapment of EN (%)	Entrapment of TA (%)	EN Release (%)	TA Release (%)
F1	800	0.6	4	38 ± 2.37	47 ± 2.84	82 ± 2.76	87 ± 3.37
F2	1000	0.1	9	43 ± 2.20	46 ± 2.21	80 ± 3.41	83 ± 3.81
F3	100	0.3	24	34 ± 1.43	37 ± 3.78	59 ± 2.68	66 ± 2.19
F4	1000	0.9	13	92 ± 2.58	94 ± 1.58	43 ± 3.75	45 ± 3.65
F5	100	0.1	24	39 ± 2.43	45 ± 2.43	81 ± 2.18	86 ± 3.90
F6	1000	0.1	24	48 ± 3.46	49 ± 2.49	54 ± 2.49	59 ± 2.89
F7	100	0.9	24	40 ± 1.65	45±1.59	63 ± 3.94	69 ± 3.65
F8	100	0.9	4	42 ± 2.27	45 ± 2.27	90 ± 4.06	93 ± 2.88
F9	300	0.5	13	52 ± 2.91	57 ± 3.91	53 ± 3.61	58 ± 2.53
F10	800	0.5	13	81 ± 1.62	86 ± 3.65	48 ± 2.99	51 ± 2.61
F11	550	0.3	4	49 ± 2.73	54 ± 2.83	63 ± 3.68	66 ± 3.89
F12	550	0.7	13	63 ± 3.23	69 ± 2.24	45 ± 2.37	48 ± 3.21
F13	550	0.3	18	84 ± 2.68	88 ± 1.85	52 ± 3.57	57 ± 2.51
F14	550	0.5	18	86 ± 3.36	90 ± 2.39	52 ± 2.82	57 ± 3.98
F15	550	0.5	9	73 ± 2.79	76 ± 2.92	60 ± 2.29	60 ± 3.95
F16	550	0.5	13	78 ± 2.29	84 ± 2.84	50 ± 4.24	53 ± 2.94
F17	550	0.5	13	80 ± 2.72	83 ± 2.62	50 ± 2.51	53 ± 3.71
F18	550	0.5	13	79 ± 3.71	83 ± 2.86	51 ± 4.85	54 ± 3.69
F19	550	0.5	13	80 ± 2.64	84 ± 2.91	50 ± 2.89	53 ± 2.73
F20	550	0.5	13	80 ± 2.82	83 ± 3.08	51 ± 2.73	53 ± 3.04

**Table 3:** ANOVA for the quadratic model and studied responses such as the entrapment and release of both drugs from the scaffold.

	PARAMETERS							
FACTORS	EN Entrapment (Y_1_)		Entrapment of TA (Y_2_)		EN Release (Y_3_)		TA Release (Y_4_)	
	*p* Value	f Value	*p* Value	f Value	*p* Value	f Value	*p* Value	f Value
Quadratic model	<0.0012	8.57	<0.0002	12.96	<0.0001	32.04	<0.0001	29.24
*x*_1_—Gelatin Concentration	0.0341	2.51	0.0158	0.8572	0.2231	1.69	0.2030	1.86
*x*_2_—Plasticizer Concentration	0.0236	1.64	0.0241	0.1524	0.6820	0.1781	0.5651	0.3539
*x*_3_—Freezing Time	0.0193	0.0829	0.0319	0.5152	0.6989	0.1585	0.6998	0.1575
*x*_1_ *x*_2_	0.6442	0.2267	0.2893	1.25	0.1695	2.19	0.3297	1.05
*x*_1_ *x*_3_	0.5716	0.3421	0.7995	0.0680	0.3546	0.9421	0.6655	0.1984
*x*_2_ *x*_3_	0.8770	0.0252	0.1808	2.07	0.9323	0.0076	0.4943	0.5032
*x*_1_^2^	0.0966	3.36	0.4516	0.6135	0.3397	1.01	0.0355	5.90
*x*_2_^2^	0.0146	8.68	0.0051	12.72	0.0001	38.08	0.0004	27.09
*x*_3_^2^	0.0321	6.37	0.0129	0.2727	0.2082	1.81	0.0306	6.33

**Table 4:** The statistics analysis of the parameters of the applied model for all studied responses.

Source	Entrapment of EN (Y_1_)	Entrapment of TA (Y_2_)	EN Release (Y_3_)	TA Release (Y_4_)
Std Deviation	6.68	6.63	4.06	4.49
Mean	60.50	56.30	71.75	65.75
C.V%	11.05	11.78	5.66	6.82
R^2^	0.8852	0.9210	0.9665	0.9634
Adjusted R^2^	0.7818	0.8500	0.9363	0.9304
Predicted R^2^	0.3484	0.3132	0.8413	0.2039
Adeq Precision	19.8629	18.7343	16.4820	14.4866

**Table 5:** Formulation components and obtained outcomes of entrapment efficiency, release of drugs, zeta potential and desirability factor of the optimized scaffold formulation.

Composition of Optimized Scaffold		Scaffold Responses	Exp. Value	Predicted Value	DF	ZP (mv)
Gelatin Conc. (mg)	1000	EN Entrapment	94	92	0.923	−16 ± 2.12
Plasticizer Conc. (mg)	0.7	TA Entrapment	95	92	0.894	
Freezing Time (hours)	18	EN Release	34	40	0.889	
		TA Release	48	40	0.917	

**Table 6:** Comparative in vivo antifungal activity and mean erythema scores of various formulations.

Sr. No.	Treatment	In Vivo Antifungal Activity		Mean Erythma Score		
		Rabbits with Positive Test/ Total Rabbits	Infected Sites/Log CFU	1st Day	7th Day	14th Day
1	Group I (control group)	6/6	3.94 ± 0.45	0	0	0
2	Group II (EN-TA suspension-treated group)	4/6	3.01 ± 0.35	2	3	3
3	Group III (scaffold loaded with MSNs-treated group)	0/6	0	0	0	0
4	Group IV (drug-loaded MSNs-treated group)	1/6	0.19 ± 0.11	0	1	0

## Data Availability

MDPI Research Data Policies at https://www.mdpi.com/ethics.
